# Distinctive Morphological Patterns of Complicated Coronary Plaques in Acute Coronary Syndromes: Insights from an Optical Coherence Tomography Study

**DOI:** 10.3390/diagnostics12112837

**Published:** 2022-11-17

**Authors:** Horea-Laurentiu Onea, Mihail Spinu, Calin Homorodean, Maria Olinic, Florin-Leontin Lazar, Mihai Claudiu Ober, Diana Stoian, Lucian Mihai Itu, Dan Mircea Olinic

**Affiliations:** 1 Medical Clinic Number 1, “Iuliu Haţieganu” University of Medicine and Pharmacy, 400012 Cluj-Napoca, Romania; 2 Department of Interventional Cardiology, Cluj County Emergency Hospital, 400006 Cluj-Napoca, Romania; 3 Advanta, Siemens SRL, 500097 Brasov, Romania; 4 Department of Automation and Information Technology, Transilvania University of Brașov, 500174 Brasov, Romania

**Keywords:** atherosclerotic coronary disease, acute coronary syndromes, optical coherence tomography, culprit lesions, plaque rupture, plaque erosion, significant stenosis, calcified nodule

## Abstract

Optical coherence tomography (OCT) is an ideal imaging technique for assessing culprit coronary plaque anatomy. We investigated the morphological features and mechanisms leading to plaque complication in a single-center observational retrospective study on 70 consecutive patients with an established diagnosis of acute coronary syndrome (ACS) who underwent OCT imaging after coronary angiography. Three prominent morphological entities were identified. Type I or intimal discontinuity, which was found to be the most common mechanism leading to ACS and was seen in 35 patients (50%), was associated with thrombus (68.6%; *p* = 0.001), mostly affected the proximal plaque segment (60%; *p* = 0.009), and had no distinctive underlying plaque features. Type II, a significant stenosis with vulnerability features (inflammation in 16 patients, 84.2%; thin-cap fibroatheroma (TCFA) in 10 patients, 52.6%) and a strong association with lipid-rich plaques (94.7%; *p* = 0.002), was observed in 19 patients (27.1%). Type III, a protrusive calcified nodule, which was found to be the dominant morphological pattern in 16 patients (22.9%), was found in longer plaques (20.8 mm vs. 16.8 mm ID vs. 12.4 mm SS; *p* = 0.04) and correlated well with TCFA (93.8%; *p* = 0.02) and inflammation (81.3%). These results emphasize the existence of a wide spectrum of coronary morphological patterns related to ACS.

## 1. Introduction

Acute coronary syndromes (ACS), the most severe expressions of coronary artery disease, remain a leading cause of morbidity and mortality worldwide despite continuous advances in acute care and primary and secondary prevention [1]. Based on in vitro studies [2], coronary atherosclerosis and plaque destabilization with subsequent thrombus formation are the main pathophysiological mechanisms in the majority of cases. There are also other non-atherosclerotic causes of ACS, such as spontaneous coronary artery dissection or spontaneous recanalization of coronary thrombus [3], which are less frequent but important to recognize as they require different therapeutic approaches.

Coronary angiography (CA) is a well-established invasive procedure used in both stable and acute settings for the assessment of the extent of coronary artery disease and the guidance of treatment strategies [4]. As no imaging technique is flawless, CA has known limitations regarding its inability to provide an accurate characterization of plaque morphology as well as a proper stenosis severity grading [4].

Different techniques currently exist for plaque characterization, ranging from non-invasive modalities, such as coronary computer tomography (CT), to intravascular imaging (intravascular ultrasound (IVUS) or optical coherence tomography (OCT)). Advances in coronary CT now allow for plaque geometrical and compositional assumptions [5,6] and it was proven that some of the features identified on CT imaging could correlate with the risk of future clinical events [7]. Nevertheless, CT interpretation is sometimes cumbersome due to certain factors, such as “the blooming’’ effect and low image resolution.

OCT, an intravascular imaging technique that utilizes light waves, has emerged as an adjuvant to CA, providing high-quality cross-sectional images of the vessel wall and luminal area with better resolution and tissue characterization, though it is at the cost of lower penetration when compared to IVUS [4,8]. By means of different backscattering and attenuation properties, each tissue component offers a specific OCT image. OCT provides invaluable insights in the setting of ACS [3,8,9,10,11,12,13,14,15,16,17,18,19,20] by allowing the exclusion of non-atherosclerotic causes [9,10,11] as well as defining the vulnerable plaque by evaluating the fibrous cap thickness and degree of macrophage infiltration [4]. Equally important, it can assess the culprit atherosclerotic lesions, with its favorable ability in detecting plaque erosion (PE), plaque rupture (PR) [21], or due to adequate calcium penetration, the calcified nodule (CN) [22]. In addition, it offers a suitable discriminating capacity between red and white thrombus [23].

The aim of this study was to investigate the morphological features of culprit coronary plaques in ACS patients, using OCT imaging. We analyzed how complicated plaques appear on the luminal interface, the underlying plaque structure, and the topography of plaque complications along the plaque length. The correlation with the clinical picture was evaluated.

## 2. Materials and Methods

### 2.1. Study Population

This was an observational retrospective study conducted in a single tertiary center in Romania: Cluj County Emergency Hospital, Department of Interventional Cardiology. Consecutive patients with an established diagnosis of ACS and an indication of invasive CA who underwent OCT imaging after angiography between January 2012 and May 2021 were included.

Informed patient consent was obtained prior to the procedure. The following exclusion criteria were applied: (1) poor image quality, (2) in-stent complication, (3) non-atherosclerotic coronary lesions, and (4) unidentifiable culprit lesions.

**Figure 1:**
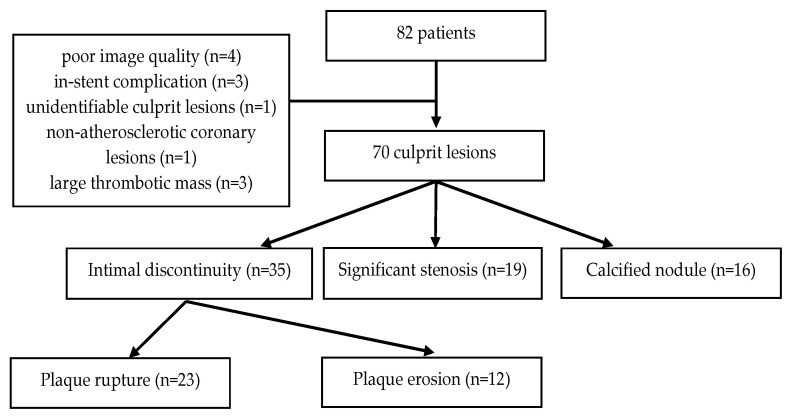
Patient flow chart.

OCT images from 82 consecutive patients were initially included. After applying the aforementioned criteria, nine patients were excluded, with three additional patients being considered ineligible due to the presence of a large thrombotic mass, which impaired lesion characterization. In total, 70 patients with 70 subsequent culprit lesions were deemed suitable for analysis (Figure 1).

Cardiovascular risk factors were defined as follows: arterial hypertension as systolic blood pressure > 140 mmHg and/or diastolic blood pressure > 90 mmHg or treated hypertension; diabetes mellitus as glycated hemoglobin level ≥ 6.5% and/or fasting glucose level ≥ 126 mg/dL or the use of anti-diabetic drugs; dyslipidemia as low-density lipoprotein cholesterol > 130 mg/dL and/or triglycerides > 150 mg/dL or treated dyslipidemia; and overweight condition as body mass index > 25 kg/m^2^.

The diagnosis of ACS included ST-segment elevation myocardial infarction (STEMI), non-ST-segment elevation myocardial infarction (NSTEMI), and unstable angina pectoris (UAP).

STEMI was defined as continuous typical chest pain that lasted more than 30 min associated with an ST-segment elevation of at least 0.1 mV in 2 or more contiguous leads or new left bundle branch block on the 12-lead electrocardiogram and elevated cardiac biomarkers (high-sensitivity cardiac troponin I, creatin kinase, and creatin kinase-MB). NSTEMI was defined as ischemic symptoms with elevated cardiac enzymes in the absence of persistent ST-segment elevation on the electrocardiogram, whereas UAP was defined as ischemic symptoms at rest in the absence of ST-segment elevation or positive cardiac biomarkers.

For the measurement of the troponin values, an Access hsTnI kit (Beckman Coulter, Brea, CA, USA) was used. This kit contains paramagnetic particles coated with mouse monoclonal anti-human cTnI antibody suspended in TRIS-buffered saline, with surfactant, bovine serum albumin (BSA), and sheep monoclonal anti-human cTnI alkaline phosphatase conjugate diluted in buffered saline, with surfactant, BSA matrix, and proteins.

The culprit lesion was confirmed based on electrocardiographic changes, echocardiographic wall motion abnormalities and angiographic appearance.

CA was performed using the existing on-site angiograph: Siemens Artis Zee (Siemens Healthineers, Erlangen, Germany).

CA and OCT imaging were performed by three senior interventional cardiologists during working hours and two days/week on call, according to the current guideline [24,25] and consensus standards [26].

### 2.2. OCT Acquisition Technique and Image Analysis

OCT uses near-infrared light (~1300 nm) to provide a tissue penetration of up to 3 mm with a high axial (10–20 µm) and lateral resolution (20–40 µm). Each OCT system consists of an imaging catheter, a drive motor operating control, and imaging software (Software version E.5.2.1, St. Jude Medical, St. Paul, MN, USA) [4].

OCT imaging was performed using a frequency-domain ILUMIEN^TM^ OPTIS^TM^ OCT system (St. Jude Medical, St. Paul, MN, USA) and C7 Dragonfly^TM^/Dragonfly^TM^ OPTIS^TM^ over-the-wire catheter (St. Jude Medical, St. Paul, MN, USA). The optical probe was manually advanced, distal to the region of interest, followed by automated pullback at a speed of 20 mm/s with simultaneous blood displacement using contrast media manually injected through the guiding catheter.

All OCT images were digitally archived in a dedicated database (RoM1OCTRegistry) and then analyzed by two independent, experienced interventional cardiologists (C.H. and M.O.), who were blinded to the patients’ clinical and paraclinical findings. Any inconsistencies between the observers were mediated by a third physician (D.M.O.). Image post-processing was employed using deep learning-based models to potentially provide an automated assessment of coronary artery disease. When the culprit lesion was identified, key morphological features were defined, as shown in Table 1.

Total plaque length was measured as the distance from diseased-to-diseased segment. Plaque anatomy was analyzed at increments of 1 mm. Each plaque was separated by a 5 mm disease-free section. Lipid-rich plaques (LRPs) and thin-cap fibroatheromas (TCFA) were defined as containing two or more quadrants of lipid pool/necrotic core. Fibrous cap thickness was represented as the average of three different measurements at the thinnest part.

This study complies with the Declaration of Helsinki on human research.

Reporting of the study conforms to the broad EQUATOR guidelines [29].

### 2.3. Statistical Analysis

Statistical analysis was performed using IBM SPSS version 26.0 (SPSS Inc, Chicago, IL, USA) from a Microsoft Excel 2019 database. Continuous variables are expressed as mean ± standard deviation, while categorical variables are expressed as counts and percentages. To study the difference between continuous variables, the Kruskal–Wallis test was used, without making any assumptions on data distribution. Pearson’s chi-squared test was performed for correlations between categorical dichotomous or multinomial variables. Fisher’s exact test was instead employed when the expected cell count in the cross-tabulation was less than 5. A *p*-Value < 0.05 was considered statistically significant.

## 3. Results

The baseline patient characteristics are summarized in Table 2. The average patient age was 59.7 years with more than two-thirds of the patients being male (65.7%). The risk profile was similar between the groups, with a high prevalence of hypertension (78.6%) and dyslipidemia (65.7%) and a low rate of smoking (20%) and diabetes mellitus (25.7%). With respect to the diagnosis at admission, a larger proportion of patients had UAP (48.6%), as compared to STEMI (24.3%) or NSTEMI (27.1%).

The main angiographic findings are presented in Table 3. The left anterior descending artery was the culprit vessel in 78.6% of cases with no differences between the groups in terms of predilection for a particular vessel (*p* = 0.64). In 44.3% of cases, culprit lesions were severely stenotic whereas more than half of the patients (55.7%) showed multivessel disease status.

**Figure 2:**
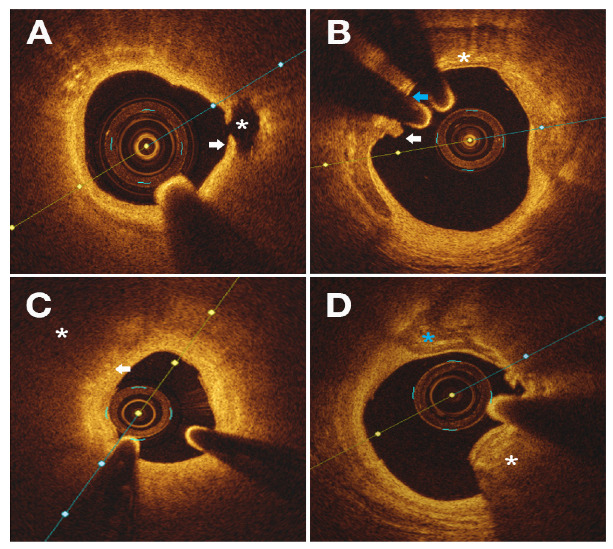
Representative OCT images of culprit lesions. (**A**) Plaque rupture with a disrupted fibrous cap (arrow) and a clear cavity (star). (**B**) Plaque erosion. White thrombus (white arrow) overlying a thin fibrous cap (blue arrow) and calcium sheets (star), with no evidence of rupture. (**C**) Significant stenosis within lipid-rich plaque (star), which exhibits signs of macrophage infiltration (arrow). (**D**) Calcified nodule (white star) with adjacent calcium sheets (blue star).

Three distinct morphological entities were identified (Figure 2). Type I, intimal discontinuity (ID), was the most common mechanism leading to ACS and was seen in 35 patients (50%), comprised of PR in 23 patients (32.9%) and PE in 12 patients (17.1%). Type II, a significant stenosis (SS) with vulnerability features, including macrophage infiltration in 16 patients (84.2%) and TCFA in 10 patients (52.6%), was present in 19 patients (27.1%). Type III, a protrusive CN, was the dominant morphological pattern in 16 patients (22.9%).

The clinical presentation of ACS is significantly correlated with plaque morphology (Table 2). Most STEMI patients presented with ID type I plaques (12/17 patients, 70.6%), while type II SS plaques without ID and type III CN only accounted for 2/17 (11.8%) and 3/17 patients (17.6%), respectively. NSTEMI patients mainly had ID type I or CN type III plaques (with 8/19 patients, 42.1%, each), while type II plaques only represented 3/19 patients (15.8%). UAP patients had as main features either a type I plaque with ID (15/34 patients, 44.1%) or a SS type II plaque without identifiable ID or thrombus (14/34 patients, 41.2%), while the CN type II plaque was present in only 5/34 of cases (14.7%).

Most of the patients underwent invasive treatment, mainly PCI (74.3%). Identification of an SS type II lesion prompted specific treatment in all cases, while a conservative management was employed in 9/35 patients (25.7%) with ID type I and 5/16 patients (31.2%) with CN type III plaques, respectively (Table 2).

**Figure 3:**
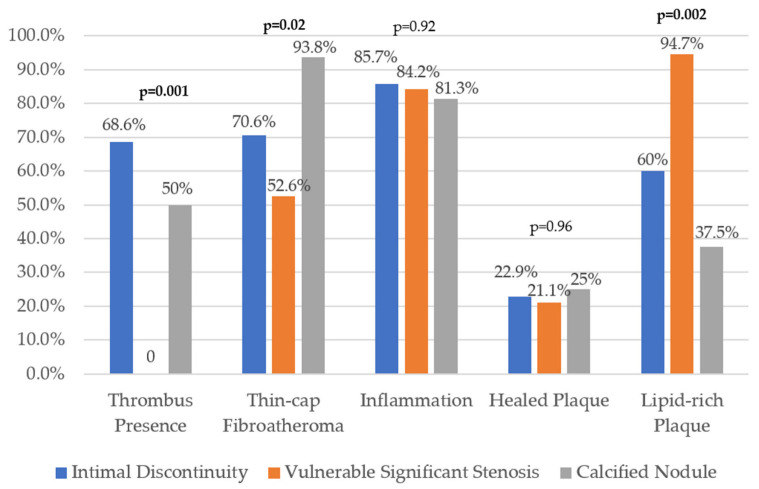
Main correlations for each patient group.

The presence of a thrombus was significantly associated with ID plaques (68.6%; *p* = 0.001) (Figure 3). TCFA was significantly more prevalent in CN plaques (93.8%; *p* = 0.02), as compared to ID type I or type II plaques. Intimal inflammation had a similarly high prevalence within the three patterns (85.7% in ID, 84.2% in SS, 81.3% in CN; *p* = 0.92). Healed plaque component prevalence was also similar across the groups (22.9% in ID, 21.1% in SS, 25% in CN; *p* = 0.96). LRPs were highly prevalent and significantly associated with SS lesions (94.7%; *p* = 0.002).

Patients with a CN had longer culprit plaques (20.8 ± 12.6 mm vs. 16.8 ± 9.3 mm in ID vs. 12.4 ± 6.7 mm in SS; *p* = 0.04) and a trend towards more severe coronary disease (75% vs. 54.3% in ID vs. 42.1% in SS; *p* = 0.06) (Table 3). A severely tight lesion (>90%) was more often found in type II SS plaques (89.5%; *p* < 0.001). In 51.4% of cases, intimal discontinuity occurred on a borderline (50–70%) lesion.

**Figure 4:**
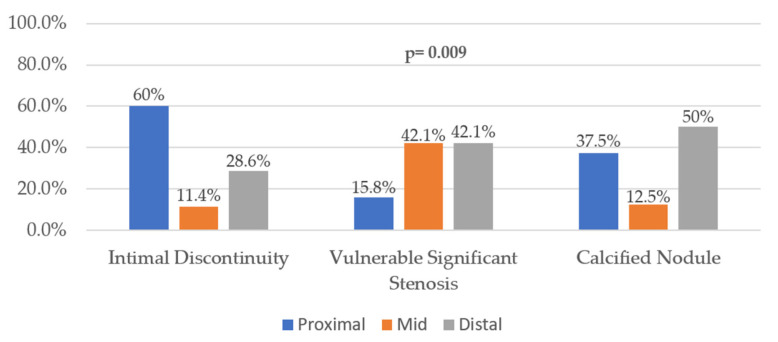
Topography of intraplaque complication.

Evaluation of the intraplaque topography of complications (Figure 4) showed that only ID had a particular pattern of occurrence, that is, in the proximal plaque segment (60%; *p* = 0.009).

**Figure 5:**
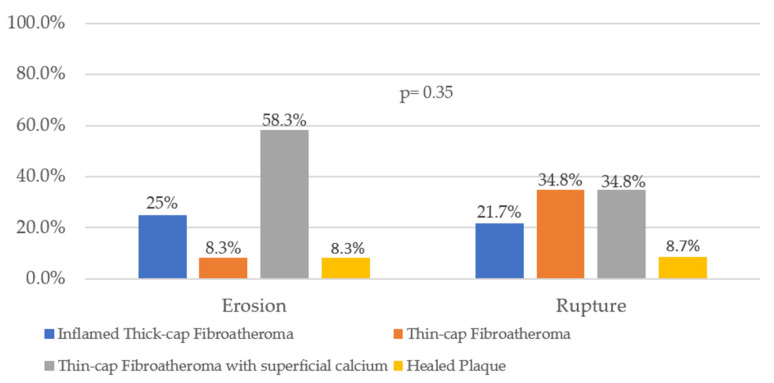
Underlying plaque configuration of intimal discontinuities.

When analyzing the underlying plaque composition for the type I entity, four main determinants of vulnerability were found: an inflamed thick-cap fibroatheroma, TCFA, TCFA flanking superficial calcium sheets, and healed plaque component (Figure 5). None of the ruptures or erosions showed a strong correlation with any of the latter (*p* = 0.35) but there was a trend towards a larger number of erosions arising in the context of TCFA with superficial calcium compared to PR (58.3% vs. 34.8%).

Regarding cardiovascular risk-lowering drugs, 51.4% of the patients were on chronic statin treatment prior to hospital admission, while 40% received aspirin. In the statin group there was a signal towards a lower number of patients manifesting ID lesions (15/35 patients, 42.9%) while more patients presented with SS type II plaques (13/19 patients, 68.4%), *p* = 0.1 (Table 2).

## 4. Discussion

In this in vivo OCT study three main morphological patterns leading to ACS were identified: ID, SS, and CN. ID was the most prevalent aspect, generally affecting the proximal plaque segment, and it was associated with the presence of a thrombotic mass and had no distinct underlying plaque features. The clinical appearance of ID plaques was mostly UAP but could also be NSTEMI or STEMI. SS lesions typically appeared on LRPs and were associated with vulnerability characteristics: inflammation and TCFA. The clinical feature of SS plaques was mainly UAP. CN were relatively prevalent and strongly correlated with TCFA, inflammation, and longer coronary plaques. The clinical feature of CN was mainly NSTEMI.

With respect to the type I entity, PR is the most prevalent cause of coronary thrombosis in patients with sudden cardiac death or fatal myocardial infarction (MI), as shown by pathology studies [30,31] (60–73% of cases). In patients presenting with acute non-fatal MI, PR was found in 66% of cases, as detected by IVUS [32], and in 44–73% of cases, as shown by OCT studies [21,22]. Moreover, it is known that the incidence of PR is higher in STEMI patients [33] (70%; *p* = 0.03), while the presence of a large thrombotic mass is mainly seen in STEMI patients and, to a smaller degree, in NSTEMI ones [33] (78% vs. 27%; *p* < 0.001).

In contrast to these findings, the incidence of PR seen in our study was lower: only 32.9%. Several factors could have accounted for this difference. Studies that found higher PR incidences [21,22,30,31,32] included more severe patients, either with sudden cardiac death and fatal MI or non-fatal MI, whereas most of our patients had UAP (48.6%). In addition, a selection bias in our research was related to the impaired imaging conditions in the presence of a large and occlusive thrombus, which determined the operators to limit the use of OCT imaging. This led to a low prevalence of STEMI and NSTEMI patients included in our study (24.3% and 27.1%, respectively). Another important aspect to consider is prior medication, as it was shown that patients on chronic statin treatment had lower incidences of PR [34]. Indeed, more than half of our cohort received statin before admission and there was a trend towards fewer ID lesions seen in these patients.

PE was identified in up to 31% of ACS cases, being the second cause of coronary plaque thrombosis [31]. In comparison, our study showed a lower incidence of 17.1% for this entity. This could be explained by the clinical status of our patients, with a high prevalence of UAP. Another possible explanation for our findings could be the particular risk profile of our patients, notably the low incidence of smoking (20%). Smoking is one of the most powerful cardiovascular risk factors with a dose-dependent effect on MI rate (eight-fold increased risk for those smoking more than 25 cigarettes per day) [35]. It was proven that smoking is associated with PE in both men and women [30]. This lower smoking risk profile may have accounted for the lower rate of PE seen in our study.

Our study has shown that ID lesions (both PE and PR) tend to occur more often in the proximal plaque segments. Similar results were obtained by Fukumoto et al. [36], by means of a three-dimensional IVUS color mapping system (used to localize areas of elevated shear stress along the plaque length), showing that proximal plaque segments are more prone to PR. In contrast, a small retrospective study [37] demonstrated PE to have mostly a distal localization (*p* = 0.01). Results should be interpreted with caution in the latter instance since in the erosion group, lesion preparation with predilatation was often used, which could have led to alterations in the plaque morphology. Moreover, removal of thrombus from the proximal segments through thromboaspiration could have arisen, thus creating a pseudo-distal erosion impression.

In the proximal part of the atherosclerotic plaques, identification of the complications leading to ACS and the fact that these plaques often are of borderline angiographic severity support the interest of OCT imaging. This could improve the detection of the proximal complicated plaque segments, which may have been missed by CA, thus resulting in the selection of longer stents to allow for optimal lesion coverage.

In our cohort, no specific underlying plaque morphology was significantly associated with the type I plaque complication profile. Our study showed that ID lesions may occur in various forms of complicated plaques, from thin- to thick-cap fibroatheroma, in superficial calcium or healed plaques. Although pathology data [31] show that PR generally occurs on TCFAs, Jia et al. [22] found that 100% of OCT-detected PR occurred on LRPs, while only 67.3% of them were TCFAs. In another study on 1660 STEMI patients [38], PR was detected on thick-cap fibroatheromas in 10.2% of cases. In the situation of PE, the substrate is different: pathological intimal thickening in 16% of cases and LRPs in 84% of cases, as demonstrated by in vitro OCT studies [39]. In vivo studies [22,38] show an incidence of 50–56% for fibrous plaque and 44–50% for LRPs (including 13.5% TCFAs).

A high macrophage content was observed in this group (85.7%), especially in the case of PR (95.4%). This could provide a potential explanation for the heterogeneity of subjacent anatomy, as intimal inflammation could increase the risk of complication even in plaques than appear more “stable”.

Our research showed a trend towards more erosions in association with TCFA overlying superficial calcium plates (58.3% vs. 34.8% PR). This suggests that superficial calcium sheets can cause micro-disruptions in the intima and lead to luminal thrombosis. Accumulation of spotty calcifications in the necrotic core close to the fibrous cap is a known risk marker of plaque vulnerability and rupture [40]. However, Costopoulos et al. [41] observed that plaque shear stress (hence, the risk for PR) is reduced when dense calcium ≥10% is present. Moreover, it was demonstrated [42] that low plaque shear stress promotes low-density lipoprotein filtration, thus being associated with plaque progression but not destabilization. Future studies are needed to fully clarify the underlying mechanisms correlating calcified plaque components and vulnerability features.

Regarding the type II entity, a significant finding that derived from our work is that an SS plaque can lead to an ACS in 27.1% of cases. This can be attributed to the fact that our cohort reflects a real-life scenario, with patients from the full ACS spectrum being included. Most of the existing studies enrolled only STEMI and NSTEMI patients. In our study, most (48%) of the ACS patients presented with UAP and it is to be noted that 73% of the UAP patients exhibited this pattern of association between severe stenosis and features of high complication risk, mainly inflammation (84.2%) and TCFA (52.6%).

Manoharan et al. [43] found that in both STEMI patients after thromboaspiration and in those with NSTEMI/UAP, the culprit lesions were at least 50% stenotic by angiographic assessment. The PROSPECT study [44] included 700 patients with ACS and demonstrated that three IVUS parameters can predict future clinical events: plaque burden >70%, minimum lumen area <4 mm^2^, and the association of TCFA. We have found a significant correlation between SS lesions and, respectively, LRPs and macrophage infiltration. Furthermore, TCFA was present in more than half of the cases. Given the fact that none of these type II lesions exhibited OCT signs of thrombosis, we may consider that other adjuvant factors could play a role in the ACS clinical picture: added epicardial vasospasm, microvascular dysfunction, or transient micro-thrombosis on high-risk plaques, with spontaneous in situ resolution or distal embolism, leaving the plaque thrombus-free. As all of our ACS patients received potent antithrombotic therapy upon admission, superficial micro-thrombosis may have undergone resolution at the time of OCT imaging.

In relation to the type III cohort, a protrusive CN emerged as an important entity in our study, with an incidence of 22.9%. Data from both pathology and OCT studies describe this morphological pattern in only 5–8% of culprit plaques of ACS patients [39,45]. It is known that the presence of coronary calcification is a sign of advanced atherosclerosis and subsequently worse clinical outcomes [46]. Our findings are consistent with this data: patients with a CN have significantly longer plaques and a trend towards more severe coronary artery disease. Moreover, CN strongly correlated with the presence of TCFA (93.8%), another hallmark of advanced disease. In a study by Sugiyama et al. [47] culprit calcified plaques amounted for only 12.7% of ACS cases and they were classified into three groups based on the pattern of calcification and the integrity of the fibrous cap: eruptive calcified nodules, superficial calcific sheet, and calcified protrusion. We believe that the superficial calcific sheet subtype defined as “sheet-like superficial calcific plate without erupted nodules or protruding mass into the lumen” may be considered equivalent to the TCFA overlying superficial calcium observed by us. Macrophage infiltration was also prevalent in this group (81.3%), which is in line with the developing process of TCFA and intimal calcification.

As our study investigated only patients with “traditional” risk factors, a particular area of interest for future research could be certain high-risk conditions associated with accelerated atherosclerosis, such as acquired immunodeficiency syndrome [48]. Shedding light on their pathological features could help in the disease management and development of certain tailored treatments.

Several limitations of this study need to be addressed. Given the small cohort size from a single-center and its retrospective nature, caution is advised when interpreting the data and they should be considered hypothesis-generating. As both clinical and imaging follow-up was beyond the scope of this investigation, its long-term significance remains to be known; nevertheless, it paves the road for future studies. Fewer STEMI and NSTEMI patients were included compared to UAP. Left anterior descending was the examined vessel in the vast majority of cases, partially due to the fact that OCT was more likely to be performed in this situation as the left anterior descending artery has prognostic implications. A small number of patients were excluded, mainly because of poor image quality, in-stent complications, and thrombus, precluding analysis of underlying plaque.

## 5. Conclusions

This study demonstrates a wide spectrum of culprit plaque morphological patterns in ACS patients, corresponding to the clinical heterogeneity of this disease. Type I or ID plaque (with PR or PE) is the most common feature (50% of cases) and mostly affects the proximal plaque segments. In patients with borderline severity angiographic stenoses, this could impact clinical practice since OCT lesion imaging could guide the physician in selecting longer stents, thus being able to provide optimal proximal lesion coverage. There are more PE in relation to TCFA overlying superficial calcium plates, a finding that could support future studies. Type II or SS plaque emerged as an important entity and was seen in 27.1% of cases. It mostly occurs in UAP patients, has an underlying LRP, and is associated with vulnerability hallmarks (intimal inflammation and TCFA). Type III or CN plaque has a prevalence higher than previously described (22.9%), is mainly found in NSTEMI patients with longer coronary plaques and more severe disease, and is strongly correlated with the presence of TCFA and macrophage infiltration.

## Figures and Tables

**Table 1:** OCT definition of main morphological patterns (adapted from Otsuka F et al. [27], Tearney GJ et al. [26], Subban V et al. [28], and Spinu M et al. [8]).

Histology	OCT Features
LRP (thick-cap FA)	High-attenuating and low signal regions with poorly delineated borders surrounded by a signal-rich layer, the fibrous cap
Fibrocalcific Plaque	Low-attenuating and low signal regions with sharply defined borders
TCFA	Large lipid/necrotic core encapsulated by a thin fibrous cap (<65 µm); superficial calcified plates were also included here
Macrophage Infiltration	High-attenuating and high signal dotted or linear structures located at the fibrous cap–necrotic core interface
Plaque Rupture	Fibrous cap discontinuity associated with a clearly defined intraplaque cavity
Plaque Erosion	Presence of thrombus or luminal irregularity without a visible rupture site
Calcified Nodule	High-attenuating and strong signal entity consisting solely of calcium that protrudes into the lumen, leading to thrombosis; a necrotic core is seldom present
Healed Plaque	Single or multiple layer aspect with different optical densities enveloping a large necrotic core; calcifications can be present
Thrombus	Intraluminal mass that is ± attached to the luminal surface
	Red thrombus—high attenuation and intense signal
	White thrombus—low attenuation and homogenous signal

OCT: Optical coherence tomography; LRP: lipid-rich plaque; FA: fibroatheroma; TCFA: thin-cap fibroatheroma.

**Table 2:** Baseline patient characteristics.

Variables	Overall (n = 70)	ID (n = 35)	SS (n = 19)	CN (n = 16)	*p*-Value
Age (years)	59.7 ± 11.5	58.1 ± 11.8	58.8 ± 9.4	64 ± 12.5	0.08
Male sex	46 (65.7)	21 (60)	12 (63.1)	13 (81.2)	0.03
CV risk factors					
Hypertension	55 (78.6)	27 (77.1)	15 (78.9)	13 (81.2)	0.66
DM	18 (25.7)	11 (31.4)	4 (21)	3 (18.8)	0.05
Dyslipidemia	46 (65.7)	23 (65.7)	14 (73.7)	9 (56.2)	0.13
Smoking habit	14 (20)	8 (22.9)	3 (15.8)	3 (18.8)	0.34
Overweight	9 (12.9)	3 (8.6)	4 (21)	2 (12.5)	0.06
Clinical presentation					
STEMI	17 (24.3)	12 (34.3)	2 (10.5)	3 (18.8)	0.04
NSTEMI	19 (27.1)	8 (22.9)	3 (15.8)	8 (50)	0.03
UAP	34 (48.6)	15 (42.9)	14 (73.7)	5 (31.2)	0.03
Prior medication					
Aspirin	28 (40)	12 (34.3)	9 (47.4)	7 (43.6)	0.6
Statin	36 (51.4)	15 (42.9)	13 (68.4)	8 (50)	0.1
Management					**0.03**
PCI	52 (74.3)	24 (68.6)	19 (94.7)	9 (56.3)	
CABG	4 (5.7)	2 (5.7)	1 (5.3)	2 (12.5)	
Conservative	14 (20)	9 (25.7)	0 (0)	5 (31.2)	

Values are mean ± SD for continuous variables and n (%) for categorical variables. ID: intimal discontinuity; SS: significant stenosis; CABG: coronary artery bypass grafting; CN: calcified nodule; CV: cardiovascular; DM: diabetes mellitus; STEMI: ST-segment elevation myocardial infarction; NSTEMI: non-ST-segment elevation myocardial infarction; PCI: percutaneous coronary intervention; UAP: unstable angina pectoris.

**Table 3:** Angiographic findings.

Variables	Overall (n = 70)	ID (n = 35)	SS (n = 19)	CN (n = 16)	*p*-Value
Culprit vessel					0.64
LM	9 (12.9)	6 (17.1)	0 (0)	3 (18.8)	0.43
LAD	55 (78.6)	25 (71.4)	17 (89.5)	13 (81.3)	0.71
LCx	2 (2.9)	1 (2.9)	1 (5.3)	0 (0)	0.78
RCA	4 (5.7)	3 (8.6)	1 (5.3)	0 (0)	0.66
Lesion severity (%)					**<0.001**
50–70	20 (28.6)	18 (51.4)	0 (0)	2 (12.5)	
70–90	19 (27.1)	7 (20)	2 (10.5)	10 (62.5)	
>90	31 (44.3)	10 (28.6)	17 (89.5)	4 (25)	
Lesion length (mm)	16.5 ± 9.9	16.8 ± 9.3	12.4 ± 6.7	20.8 ± 12.6	**0.04**
Multivessel disease *	39 (55.7)	19 (54.3)	8 (42.1)	12 (75)	0.06

Values are mean ± SD for continuous variables and n (%) for categorical variables. ID: intimal discontinuity; SS: significant stenosis; CN: calcified nodule; LM: left main stem; LAD: left anterior descending coronary artery; LCx: left circumflex artery; RCA: right coronary artery. * Significant (>70% quantitative coronary angiography) stenosis in any of the non-culprit vessels or LM disease.

## Data Availability

The data presented in this study are available on request from the corresponding author. The data are not publicly available because they are property of Cluj County Emergency Hospital, Cluj-Napoca, Romania.
